# Supplementary material for: Land snails of Leptopoma Pfeiffer, 1847 in Sabah, Northern Borneo (Caenogastropoda: Cyclophoridae): an analysis of molecular phylogeny and geographical variations in shell form
Source: PeerJ. 2017 Oct 31;5:e3981. doi: 10.7717/peerj.3981 (PMC5669252; doi:10.7717/peerj.3981)
Supplement: File S2 — Table S1. Specimens information. Table S2. Shell colour patterns description. Table S3 & S4. Results normality tests and homogeneity of variances tests prior to ANOVA. Table S5 & S6. Frequency data of shell qualitative characters used for chi-square tests. [file peerj-05-3981-s002.docx]

SUPPLEMENTARY FILE 2

**Table S1** List of specimens (species, voucher ID, locality, latitude and longitude) and GenBank accession number for both COI and 16S sequences.

|  | **Species** | **Voucher ID** | **Locality (Country, State, Location)** | **Latitude**  **(°)** | **Longitude**  **(°)** | **GenBank Accession No.** | | |
| --- | --- | --- | --- | --- | --- | --- | --- | --- |
|  |  |  |  |  |  | **COI** | **16S** | **ITS-1** |
| 1 | *L. sericatum* | BOR/MOL 5963 | Malaysia, Sabah, Sipitang, Muaya | 4.926 | 115.691 | N/A | KR736309 | MF414138 |
| 2 | *L. sericatum* | BOR/MOL 5968 | Malaysia, Sarawak, Sibu, Kapit, Lanjak-Entimau WS. LEWS expedition. | 1.706 | 112.589 | KR736102 | KR736307 | MF414132 |
| 3 | *L. sericatum* | BOR/MOL 5969 | Malaysia, Sarawak, Sibu, Kapit, Lanjak-Entimau WS. LEWS expedition. | 1.706 | 112.589 | KR736101 | KR736306 | N/A |
| 4 | *L. sericatum* | BOR/MOL 5970 | Malaysia, Sarawak, Sibu, Kapit, Lanjak-Entimau WS. LEWS expedition. | 1.706 | 112.589 | KR736100 | KR736305 | N/A |
| 5 | *L. sericatum* | BOR/MOL 5980 | Malaysia, Sabah, Lahad Datu, Tabin Wildlife Reserve. | 5.014 | 118.392 | KR736107 | KR736314 | N/A |
| 6 | *L. sericatum* | BOR/MOL 5981 | Malaysia, Sabah, Lahad Datu, Tabin Wildlife Reserve. | 5.014 | 118.392 | N/A | KR736313 | MF414144 |
| 7 | *L. sericatum* | BOR/MOL 5982 | Malaysia, Sabah, Lahad Datu, Tabin Wildlife Reserve. | 5.014 | 118.392 | KR736106 | KR736312 | MF414143 |
| 8 | *L. sericatum* | BOR/MOL 5984 | Malaysia, Sabah, Tambunan, Gunung Alab | 5.830 | 116.341 | KR736105 | KR736311 | MF414140 |
| 9 | *L. sericatum* | BOR/MOL 5964 | Malaysia, Sabah, Lower Kinabatangan | 5.532 | 118.070 | KR736103 | KR736308 | MF414136 |
| 10 | *L. sericatum* | BOR/MOL 5978 | Malaysia, Sabah, Nabawan, Persiangan. Sapulut. Batu Sanaron (outside cave) | 4.673 | 116.566 | KR736099 | KR736304 | MF414142 |
| 11 | *L. pellucidum* | BOR/MOL 5976 | Malaysia, Sabah, Kota Kinabalu, Pulau Manukan | 5.976 | 116.001 | KR736095 | KR736300 | MF414141 |
| 12 | *L. pellucidum* | BOR/MOL 5994 | Malaysia, Sabah, Kota Kinabalu, UMS hill, Behind ITBC. | 6.042 | 116.118 | KR736097 | KR736302 | MF414139 |
| 13 | *L. pellucidum* | BOR/MOL 6009 | Malaysia, Sabah, Kota Belud, Pulau Mantanani Besar | 6.711 | 116.355 | KR736096 | KR736301 | MF414137 |
| 14 | *L. pellucidum* | BOR/MOL 6014 | Malaysia, Sabah, Kuala Penyu, Pulau Tiga | 5.725 | 115.652 | KR736094 | KR736299 | MF414134 |
| 15 | *L. pellucidum* | BOR/MOL 5992 | Malaysia, Sabah, Kota Kinabalu, UMS hill, Behind ITBC. | 6.042 | 116.118 | KR736098 | KR736303 | MF414131 |
| 16 | *L. pellucidum* | BOR/MOL  6063 | Silam Coast Conservation Area, SC trail 1 | 4.919 | 118.179 | KU986304 | KU905047 | N/A |
| 17 | *L. pellucidum* | BOR/MOL 6064 | Mengalum Island Plot 2 | 6.190 | 115.596 | KU986305 | KU905048 | MF414135 |
| 18 | *L. atricapillum* | BOR/MOL 6070 | Silam Coast Conservation Area, Pandanus beach. | 4.919 | 118.179 | KU986303 | KU905046 | N/A |
| 19 | *L. atricapillum* | BOR/MOL 6068 | Silam Coast Conservation Area, Serangga Island. | 4.925 | 118.201 | KU986303 | KU905045 | MF414129 |
| 20 | *L. undatum* | BOR/MOL 6031 | Sugut Forest Reserve. Rose Station. | 6.387 | 117.549 | MF414128 | KR736315 | MF414130 |
| 20 | *L. tigris*¹ |  | Taiwan |  |  | EU219784.1 | EU219825.1 | N/A |
| 21 | *L. vitreum*² |  | Thailand |  |  | KJ407265.1 | KJ407187.1 | N/A |
| 22 | *Cyclophorus formosensis*¹ |  | Taiwan |  |  | EU219740.1 | EU219799.1 | N/A |

¹Lee *et al*., 2008a; ²Nantarat *et al*., 2014

**Table S2** Detail description of shell patterns.

| **Shell patterns** | **Description** |
| --- | --- |
| P1 | Shell white, unbanded |
| P2 | Shell white, with two thick and often brown spiral bands, one above periphery and another below periphery |
| P3 | Many thin spiral bands following the prominent spiral sculpture |
| P4 | Mottled of brown colouring |
| P5 | Shell white, with one thick and often brown spiral band below periphery |
| P6 | Many thin spiral bands with one thick bands above periphery |
| P7 | Many thin spiral bands with one thick bands below periphery |
| P8 | Mottled with one thick band below periphery |

**Table S3** p-value for normality of dataset from Shapiro-Wilk test. Bolded values indicated non-normality.

|  | **BI-pellucidum** | **K-pellucidum** | **BI- sericatum** | **K-sericatum** |
| --- | --- | --- | --- | --- |
| **SH** | 0.217 | 0.183 | 0.312 | **0.016** |
| **SW** | 0.708 | **0.035** | 0.283 | **0.036** |
| **AH** | 0.190 | **0.041** | 0.261 | 0.507 |
| **AW** | 0.462 | 0.310 | **0.000** | **0.030** |
| **SpH** | 0.132 | **0.020** | 0.073 | 0.089 |
| **SH/SW** | 0.612 | **0.001** | 0.418 | **7.979e-05** |

**Table S4** p-value of homogeneity of variance from Levene’s test. Bolded values indicated non-homogeneity of variances.

|  | ***L. pellucidum* from both locations** | ***L. sericatum* from both locations** | **Balambangan Island** | **Kinabatangan** |
| --- | --- | --- | --- | --- |
| **SH** | 0.173 | **0.019** | 0.102 | 0.371 |
| **SW** | 0.082 | **0.000** | 0.199 | 0.783 |
| **AH** | 0.775 | **0.03** | 0.095 | 0.966 |
| **AW** | 0.912 | **0.013** | 0.112 | 0.418 |
| **SpH** | 0.303 | 0.627 | 0.307 | 0.691 |
| **SH/SW** | 0.379 | 0.095 | 0.085 | 0.364 |

**Table S5** Contingency table of eight shell patterns and geographical clusters for *Leptopoma pellucidum* and *Leptopoma sericatum*. The values in the brackets, () indicates expected value.

| **Shell**  **patterns** | **Balambangan Island** | **Kinabatangan** | **Total** |
| --- | --- | --- | --- |
| P1 | 45  (50) | 91  (86) | 136 |
| P2 | 2  (1) | 0  (1) | 2 |
| P3 | 31  (26) | 39  (44) | 70 |
| P4 | 7  (4) | 5  (8) | 12 |
| P5 | 1  (0) | 0  (1) | 1 |
| P6 | 2  (7) | 18  (13) | 20 |
| P7 | 0  (2) | 5  (3) | 5 |
| P8 | 3  (1) | 0  (2) | 3 |
| **Total** | **91** | **158** | **249** |

**Table S6** Contingency table of the presence of dark ring band and geographical clusters for *Leptopoma pellucidum* and *Leptopoma sericatum*. The signs 0 and 1 indicate the absence and presence of dark ring band in the aperture respectively. The values in the brackets, () indicates expected value.

| **Dark ring band** | **Balambangan Island** | **Kinabatangan** | **Total** |
| --- | --- | --- | --- |
| 0 | 91  (89) | 153  (155) | 244 |
| 1 | 0  (2) | 5  (3) | 5 |
| **Total** | **91** | **158** | **249** |
